# Thyroid diseases and risk of peripheral neuropathy in a large population-based cohort: evidence from the UK Biobank

**DOI:** 10.3389/fendo.2026.1828737

**Published:** 2026-05-19

**Authors:** Yuyu Luo, Gongxiong Yao, Chenghui Song, Chun Liao, Hongchao Zhang, Cong Liao, Xueren Ao

**Affiliations:** 1 Department of General Surgery, The Third Affiliated Hospital of Guangzhou University of Chinese Medicine, Guangzhou, China; 2 The Third Clinical Medical College of Guangzhou University of Chinese Medicine, Guangzhou, China; 3 Minimally Invasive Center for Hepatobiliary and Pancreatic Surgery, The Second Affiliated Hospital of Guangzhou University of Chinese Medicine, Zhuhai Hospital of Guangdong Provincial Hospital of Chinese Medicine, Zhuhai, China

**Keywords:** hyperthyroidism, hypothyroidism, peripheral neuropathy, thyroid diseases, UK biobank

## Abstract

**Background:**

Thyroid dysfunction has been linked to several neurological disorders, but the association between thyroid diseases and peripheral neuropathy remains insufficiently explored in large population-based cohorts. We aim to investigate the association between thyroid diseases and peripheral neuropathy.

**Methods:**

We analyzed 498,417 complete-case participants from the UK Biobank with linked hospital diagnosis records. Thyroid diseases were defined using ICD-10 codes, including hypothyroidism, hyperthyroidism, and thyroid cancer. Peripheral neuropathy was identified using ICD-10 codes G60–G64. Multivariable logistic regression models were used to estimate odds ratios (ORs) and 95% confidence intervals (CIs).

**Results:**

Peripheral neuropathy was identified in 6,341 participants. In the fully adjusted model including demographic, lifestyle, socioeconomic, and clinical covariates, hypothyroidism was significantly associated with peripheral neuropathy (OR 1.29, 95% CI 1.18–1.40), and hyperthyroidism showed a weaker but statistically significant association (OR 1.22, 95% CI 1.01–1.45). Thyroid cancer was not significantly associated with peripheral neuropathy (OR 1.16, 95% CI 0.66–1.89). In sensitivity analyses excluding participants with diabetes, hypothyroidism remained associated with peripheral neuropathy, whereas the association for hyperthyroidism was attenuated.

**Discussion:**

Clinically diagnosed hypothyroidism and hyperthyroidism were associated with higher odds of peripheral neuropathy in this UK Biobank-based analysis. These findings should be interpreted as associations rather than causal relationships, given the observational design and lack of thyroid hormone levels, disease severity, and treatment information.

## Introduction

Peripheral neuropathy is a common neurological disorder characterized by damage to peripheral nerves and can lead to sensory loss, pain, and functional impairment (1, 2). The condition affects millions of individuals worldwide and is associated with significant morbidity and reduced quality of life (3, 4). Diabetes mellitus is widely recognized as the most frequent cause of peripheral neuropathy (5, 6), but a number of other metabolic and systemic conditions may also contribute to nerve dysfunction.

Thyroid diseases are among the most common endocrine disorders in the general population (7). Both hypothyroidism and hyperthyroidism are known to influence multiple organ systems, including the nervous system (8, 9). Previous clinical studies have reported neurological manifestations associated with thyroid dysfunction, such as neuropathy, myopathy, and cognitive impairment (10). For example, hypothyroidism has been linked to peripheral nerve slowing and sensory neuropathy (11), while hyperthyroidism may affect neuromuscular function through metabolic and hormonal alterations (12). However, most existing evidence is derived from relatively small clinical studies or case series, and population-based evidence remains limited.

Large-scale epidemiological studies examining the relationship between thyroid diseases and peripheral neuropathy are still limited. In particular, the potential associations of different thyroid conditions, including hypothyroidism, hyperthyroidism, and thyroid cancer, with peripheral neuropathy have not been systematically investigated in large population cohorts. Understanding these associations may help clarify whether thyroid dysfunction contributes to neuropathy risk beyond well-established factors such as diabetes. The UK Biobank provides a unique opportunity to examine these relationships in a large population-based cohort with detailed health records. Therefore, in this study, we investigated the association between thyroid diseases and the risk of peripheral neuropathy using data from nearly half a million participants in the UK Biobank. We further evaluated the robustness of the findings through multivariable adjustment, subgroup analyses, and sensitivity analyses.

## Methods

### Study population

This study was conducted using data from the UK Biobank, a large population-based cohort that recruited more than 502,129 participants aged 40–69 years across the United Kingdom between 2006 and 2010. At baseline, participants attended assessment centers where demographic characteristics, lifestyle factors, and medical history were collected through standardized questionnaires and physical examinations. The UK Biobank database is also linked to hospital admission records, which allows disease diagnoses to be identified using standardized coding systems. For the present analysis, participants with available information on thyroid diseases, peripheral neuropathy, and relevant covariates were included. Individuals with missing data on key variables were excluded. After exclusions, 499,024 participants remained in the final analysis. The complete-case analysis included 498,417 participants. Because complete diagnosis dates were not available for all peripheral neuropathy cases, the primary analysis was based on ICD-10-defined disease status using multivariable logistic regression. The UK Biobank study was approved by the North West Multi-centre Research Ethics Committee, and all participants provided written informed consent.

### Assessment of thyroid diseases

Thyroid diseases were identified using hospital diagnosis records based on the International Classification of Diseases, Tenth Revision (ICD-10). Hypothyroidism was defined using the ICD-10 code E03, while hyperthyroidism was defined using the ICD-10 code E05. Thyroid cancer was identified using the ICD-10 code C73. Participants with any of these diagnostic codes were classified accordingly, and individuals without these diagnoses were considered to have no thyroid disease. In addition to evaluating each thyroid condition separately, Hypothyroidism, hyperthyroidism, and thyroid cancer were analyzed as separate exposure variables, with participants without the corresponding thyroid disease used as the reference group. Thyroid disease status was defined using ICD-10 diagnosis codes rather than thyroid hormone measurements; therefore, biochemical severity and treatment response could not be assessed.

### Definition of peripheral neuropathy

Peripheral neuropathy was defined using hospital admission records according to ICD-10 codes G60–G64, including hereditary and idiopathic neuropathy, inflammatory polyneuropathy, other polyneuropathies, polyneuropathy in diseases classified elsewhere, and other disorders of the peripheral nervous system. The ICD-10 codes used to define peripheral neuropathy are listed in **Supplementary Table 1**. Diagnoses were identified from hospital admission records, which primarily capture moderate-to-severe conditions requiring inpatient care.

### Covariates

Covariates were selected based on clinical relevance and previous literature. Model 1 was adjusted for age and sex. Model 2 was further adjusted for body mass index (BMI), alcohol intake, smoking status, and Townsend deprivation index. Model 3 was additionally adjusted for diabetes, hypertension, chronic kidney disease, cardiovascular disease, vitamin B12 deficiency or nutritional anemia, and autoimmune diseases.

### Statistical analysis

Baseline characteristics were summarized according to thyroid disease status. Multivariable logistic regression models were used to estimate odds ratios (ORs) and 95% confidence intervals (CIs). Model 1 was adjusted for age and sex. Model 2 was further adjusted for BMI, alcohol intake, smoking status, and Townsend deprivation index. Model 3 was additionally adjusted for diabetes, hypertension, chronic kidney disease, cardiovascular disease, vitamin B12 deficiency or nutritional anemia, and autoimmune diseases. Sensitivity analyses were performed after excluding participants with diabetes. Although time-to-event analyses were considered, complete and reliable diagnosis dates were not available for all peripheral neuropathy cases in the dataset. Therefore, a full survival analysis could not be performed without introducing substantial selection bias. All statistical analyses were performed using R software (4.3.2), and a two-sided P value less than 0.05 was considered statistically significant.

## Results

### Baseline characteristics

**Figure 1:**
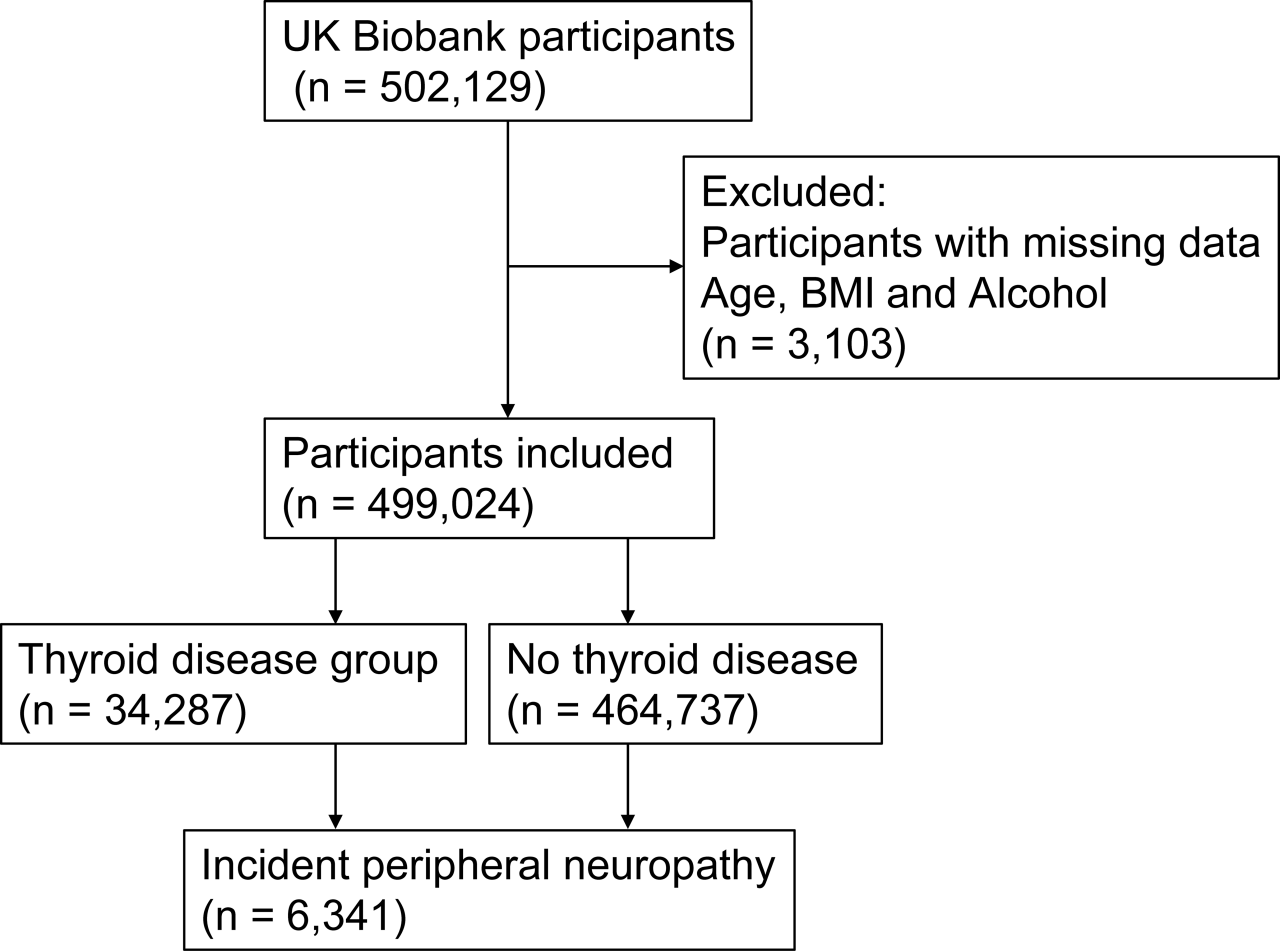
Flowchart of participant selection in the present study.

A total of 499,024 participants were included in the present analysis, including 34,287 individuals with thyroid diseases and 467,841 without thyroid diseases. Participants with thyroid diseases were older than those without thyroid diseases (58.85 ± 7.43 vs 56.36 ± 8.12 years, P<0.001) and had a higher body mass index (28.62 ± 5.53 vs 27.35 ± 4.73 kg/m², P<0.001). The proportion of males was markedly lower in the thyroid disease group compared with the non-thyroid disease group (21.3% vs 47.4%, P<0.001). Alcohol intake patterns also differed significantly between the two groups (P<0.001) (**Table 1**, **Figure 1**).

**Table 1:** Baseline characteristics of participants by thyroid disease status.

Variable	No thyroid disease (n=467,841)	Thyroid disease (n=34,287)	P value
Age, years (mean ± SD)	56.36 ± 8.12	58.85 ± 7.43	<0.001
Male, n (%)	221,680 (47.4%)	7,293 (21.3%)	<0.001
BMI, kg/m² (mean ± SD)	27.35 ± 4.73	28.62 ± 5.53	<0.001
Alcohol intake			<0.001
Daily or almost daily	96,723 (20.7%)	4,992 (14.6%)	
Never	36,286 (7.8%)	4,309 (12.6%)	
Once or twice a week	120,807 (25.8%)	8,377 (24.4%)	
One to three times a month	51,293 (11.0%)	4,519 (13.2%)	
Special occasions only	52,058 (11.1%)	5,908 (17.2%)	
Three or four times a week	109,283 (23.4%)	6,073 (17.7%)	

Continuous variables are presented as mean ± standard deviation (SD), and categorical variables are presented as number (percentage).

### Association between thyroid diseases and peripheral neuropathy

**Figure 2:**
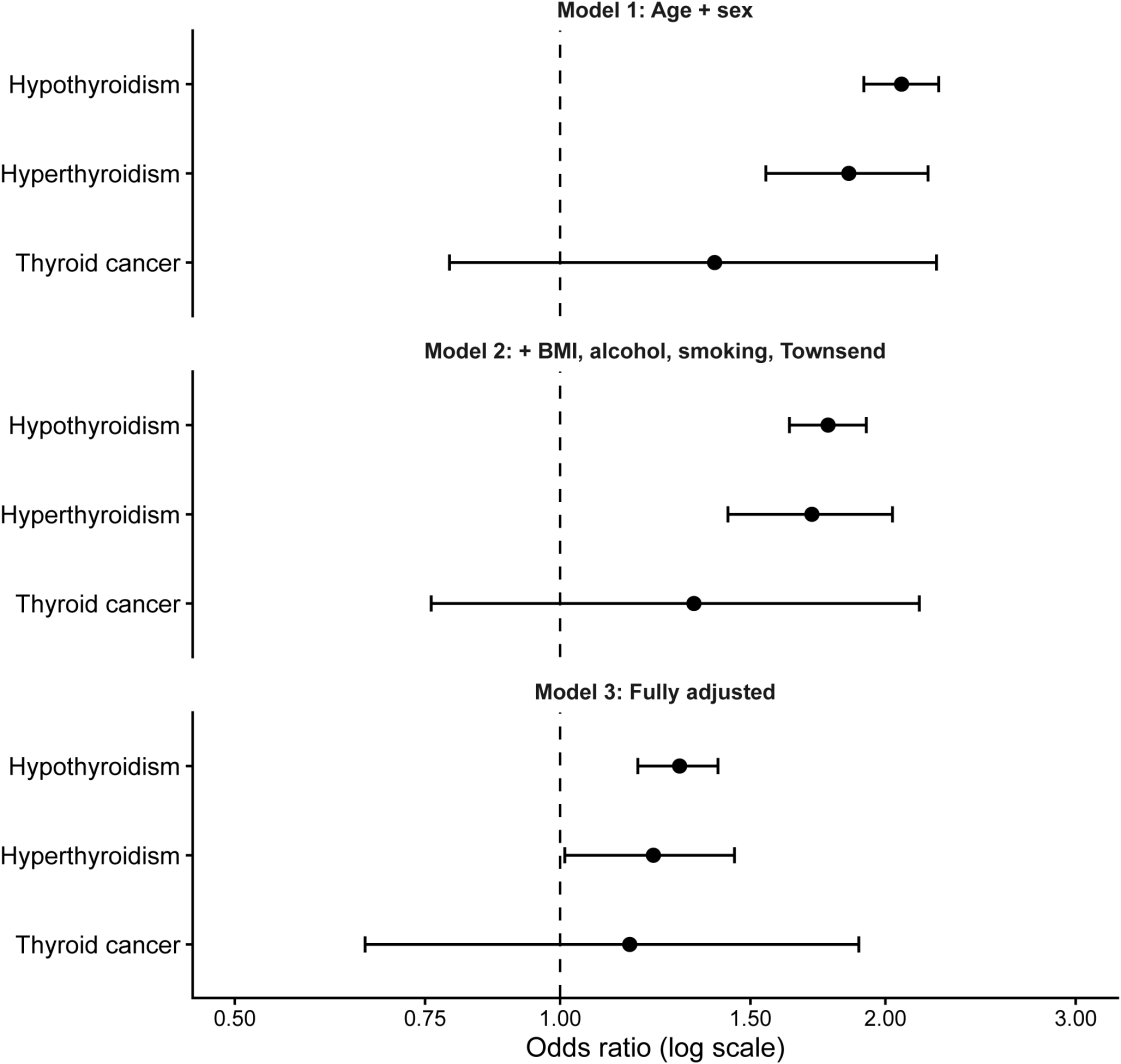
Association between thyroid diseases and peripheral neuropathy across multivariable models. Odds ratios were estimated using logistic regression models. Model 1 adjusted for age and sex; Model 2 additionally adjusted for BMI, alcohol intake, smoking status, and Townsend deprivation index; Model 3 additionally adjusted for diabetes, hypertension, chronic kidney disease, cardiovascular disease, vitamin B12 deficiency or nutritional anemia, and autoimmune diseases.

During the study period, 6,341 participants were identified with peripheral neuropathy among 498,417 complete-case participants. In Model 1 adjusted for age and sex, both hypothyroidism and hyperthyroidism were associated with higher odds of peripheral neuropathy. After further adjustment for BMI, alcohol intake, smoking status, and Townsend deprivation index, the associations remained significant for hypothyroidism (OR 1.77, 95% CI 1.63–1.92) and hyperthyroidism (OR 1.71, 95% CI 1.43–2.03). In the fully adjusted model additionally including diabetes, hypertension, chronic kidney disease, cardiovascular disease, vitamin B12 deficiency or nutritional anemia, and autoimmune diseases, hypothyroidism remained significantly associated with peripheral neuropathy (OR 1.29, 95% CI 1.18–1.40), while the association for hyperthyroidism was attenuated but remained statistically significant (OR 1.22, 95% CI 1.01–1.45). Thyroid cancer was not significantly associated with peripheral neuropathy in any model (**Table 2**, **Figure 2**). Thyroid cancer cases were relatively uncommon in the analytic cohort (n = 771), which limited the precision of the association estimate.

**Table 2:** Association between thyroid diseases and peripheral neuropathy across multivariable models.

Exposure	Model 1 OR (95% CI)	P value	Model 2 OR (95% CI)	P value	Model 3 OR (95% CI)	P value
Hypothyroidism	2.07 (1.91–2.24)	<0.001	1.77 (1.63–1.92)	<0.001	1.29 (1.18–1.40)	<0.001
Hyperthyroidism	1.85 (1.55–2.19)	<0.001	1.71 (1.43–2.03)	<0.001	1.22 (1.01–1.45)	0.034
Thyroid cancer	1.39 (0.79–2.23)	0.214	1.33 (0.76–2.15)	0.274	1.16 (0.66–1.89)	0.579

Model 1 was adjusted for age and sex. Model 2 was further adjusted for body mass index, alcohol intake, smoking status, and Townsend deprivation index. Model 3 was additionally adjusted for diabetes, hypertension, chronic kidney disease, cardiovascular disease, vitamin B12 deficiency or nutritional anemia, and autoimmune diseases. OR, odds ratio; CI, confidence interval.

### Subgroup analyses

**Figure 3:**
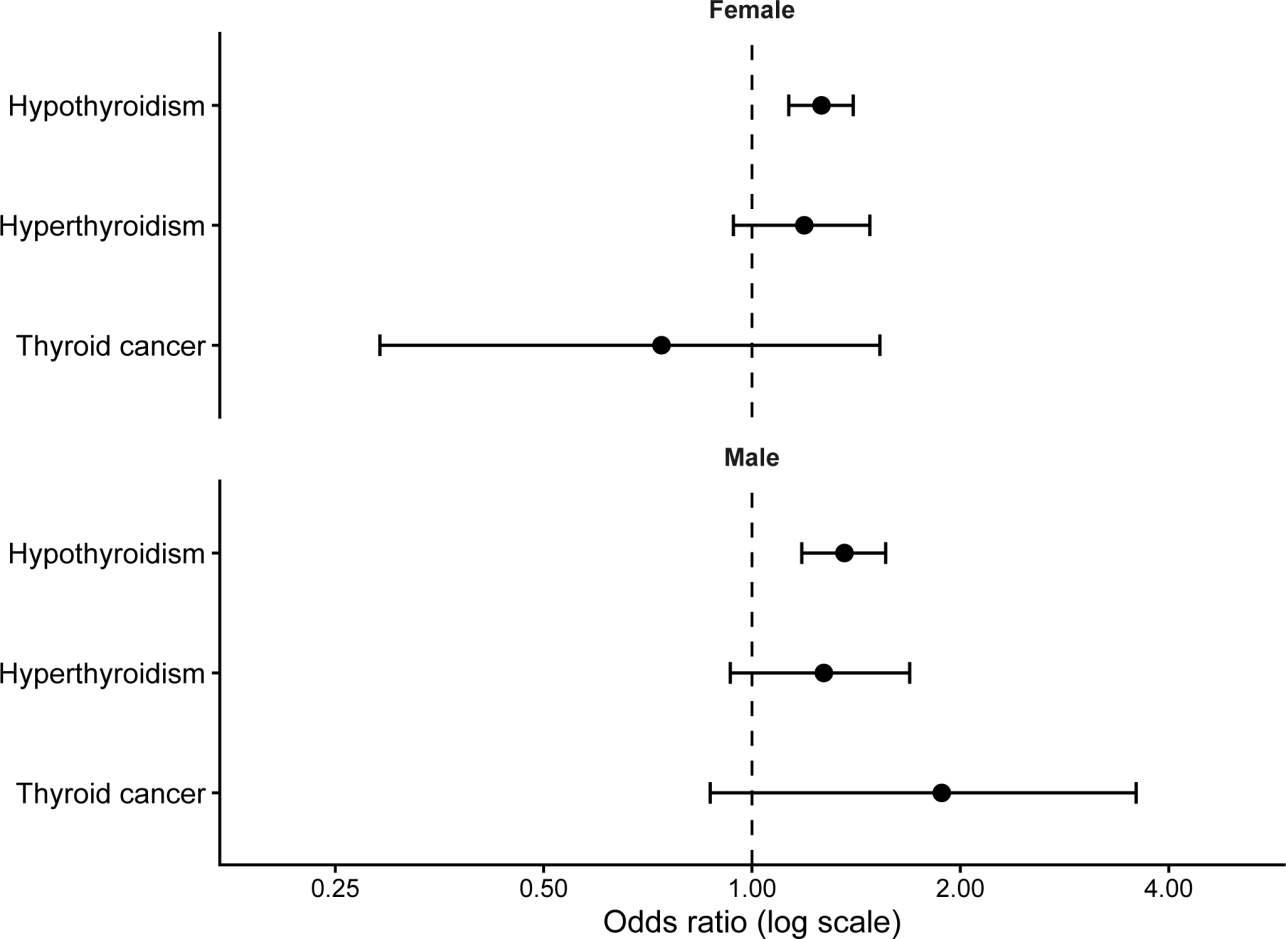
Sex-stratified association between thyroid diseases and peripheral neuropathy. Odds ratios were estimated using fully adjusted logistic regression models stratified by sex.

In sex-stratified analyses, hypothyroidism remained significantly associated with peripheral neuropathy in both females (OR 1.26, 95% CI 1.13–1.40) and males (OR 1.36, 95% CI 1.18–1.56). No significant interaction between hypothyroidism and sex was observed (P for interaction = 0.260). Associations for hyperthyroidism and thyroid cancer were not statistically significant in either sex subgroup (**Table 3**, **Figure 3**).

**Table 3:** Subgroup analysis by sex.

Exposure	Female OR (95% CI)	P value	Male OR (95% CI)	P value	P for interaction
Hypothyroidism	1.26 (1.13–1.40)	<0.001	1.36 (1.18–1.56)	<0.001	0.260
Hyperthyroidism	1.19 (0.94–1.48)	0.137	1.27 (0.93–1.69)	0.119	–
Thyroid cancer	0.74 (0.29–1.53)	0.468	1.88 (0.87–3.59)	0.078	–

Sex-stratified models were adjusted for age, body mass index, alcohol intake, smoking status, Townsend deprivation index, diabetes, hypertension, chronic kidney disease, cardiovascular disease, vitamin B12 deficiency or nutritional anemia, and autoimmune diseases. P for interaction was estimated using multiplicative interaction terms in the fully adjusted model. Interaction P values for hyperthyroidism and thyroid cancer were not estimated because of sparse events in sex-stratified categories.

### Sensitivity analysis

Sensitivity analyses were conducted after excluding participants with diabetes to evaluate whether the observed associations were driven by diabetic neuropathy. After excluding participants with diabetes, hypothyroidism remained associated with peripheral neuropathy (OR 1.51, 95% CI 1.35–1.68), whereas the association for hyperthyroidism was attenuated and no longer statistically significant (OR 1.20, 95% CI 0.92–1.53). Thyroid cancer remained nonsignificant (**Table 4**).

**Table 4:** Sensitivity analysis excluding participants with diabetes.

Exposure	OR (95% CI)	P value
Hypothyroidism	1.51 (1.35–1.68)	<0.001
Hyperthyroidism	1.20 (0.92–1.53)	0.163
Thyroid cancer	1.53 (0.73–2.79)	0.211

The model was adjusted for age, sex, body mass index, alcohol intake, smoking status, Townsend deprivation index, hypertension, chronic kidney disease, cardiovascular disease, vitamin B12 deficiency or nutritional anemia, and autoimmune diseases after excluding participants with diabetes. OR, odds ratio; CI, confidence interval.

## Discussion

In this large population-based cohort study involving nearly half a million participants, we found that both hypothyroidism and hyperthyroidism were associated with higher odds of peripheral neuropathy. These associations remained statistically significant after adjustment for demographic, lifestyle, socioeconomic, and clinical factors. In contrast, thyroid cancer was not significantly associated with peripheral neuropathy.

Peripheral neuropathy is a multifactorial neurological disorder, and metabolic conditions are known to play an important role in its development (13). While diabetes is widely recognized as the most common cause of peripheral neuropathy (14, 15), other endocrine disorders may also contribute to nerve dysfunction. Thyroid hormones are involved in multiple physiological processes, including metabolic regulation, mitochondrial function, and neuronal development (16, 17). Disturbances in thyroid hormone levels may therefore affect the peripheral nervous system through several mechanisms (18–20). Thyroid hormone imbalance may influence peripheral nerve metabolism, mitochondrial function, and axonal transport, which may help explain the observed associations.

Previous clinical studies have reported neurological manifestations associated with thyroid dysfunction (21, 22). Hypothyroidism has been linked to sensory neuropathy and slowed nerve conduction, which may be related to metabolic changes, myxedematous tissue deposition, or impaired axonal transport (23, 24). Hyperthyroidism has also been associated with neuromuscular abnormalities, possibly through increased metabolic demand and alterations in neuromuscular transmission (25, 26). However, most previous evidence has been derived from relatively small clinical studies or case series, and large population-based investigations remain limited. Our findings provide further evidence supporting a potential relationship between thyroid dysfunction and peripheral neuropathy in a large cohort setting.

The existing literature on thyroid dysfunction and peripheral neuropathy remains heterogeneous. Some studies have also reported limited or nonsignificant associations between thyroid dysfunction and peripheral neuropathy in unselected populations, highlighting the inconsistency of existing epidemiological evidence. Several small clinical and electrophysiological studies have reported sensory or sensorimotor neuropathy in patients with hypothyroidism, while other studies suggest that routine thyroid testing has relatively low diagnostic yield in unselected peripheral neuropathy evaluations. Recent genetic evidence from Mendelian randomization has also suggested potential links between hypothyroidism and several peripheral neuropathy-related outcomes, although findings differed across neuropathy subtypes. In patients with diabetes, thyroid hormone sensitivity has been associated with diabetic peripheral neuropathy, further suggesting a possible endocrine-neurological link. However, most available studies are limited by modest sample size, selected clinical populations, cross-sectional design, or focus on specific neuropathy subtypes. Therefore, our study adds population-scale evidence while also highlighting the need for further studies with detailed thyroid biomarkers, neuropathy phenotyping, and longitudinal follow-up.

Interestingly, the associations between thyroid diseases and peripheral neuropathy were observed in both males and females, although the magnitude of the associations appeared slightly stronger in males (27, 28). The reasons for these differences are not entirely clear, but sex-related differences in hormonal regulation, metabolic profiles, or healthcare utilization may play a role. Further studies are needed to clarify whether sex modifies the relationship between thyroid dysfunction and peripheral nerve disorders (29, 30). A formal time-to-event analysis was not performed because complete and reliable diagnosis dates were not available for all peripheral neuropathy cases. Therefore, logistic regression was used as the primary analytical approach.

Several strengths of this study should be noted. First, the analysis was based on a very large population-based cohort, which provided substantial statistical power to detect associations between thyroid diseases and peripheral neuropathy. Second, multiple analyses were conducted to evaluate the robustness of the findings, including adjustment for diabetes, subgroup analyses, and sensitivity analyses. These analyses consistently supported the main results.

Several limitations should be considered. First, thyroid diseases and peripheral neuropathy were identified using hospital ICD-10 codes. This approach may lead to misclassification bias, particularly because mild or subclinical cases managed in outpatient or primary care settings may not be captured. In addition, peripheral neuropathy was defined using a heterogeneous group of ICD-10 codes, and we were unable to distinguish specific neuropathy subtypes, such as diabetic, inflammatory, or hereditary neuropathies. Second, information on thyroid hormone levels, including TSH, free T4, and free T3, was not available in the current analysis. Therefore, we could not evaluate disease severity, biochemical thyroid status, or dose-response relationships between thyroid hormone abnormalities and peripheral neuropathy. Similarly, information on thyroid-related treatment, treatment duration, and biochemical control was not incorporated. Treatment status may act as both a source of residual confounding and a potential effect modifier, because adequately treated thyroid disease may differ from untreated or poorly controlled disease. Third, although we expanded covariate adjustment to include smoking status, socioeconomic deprivation, diabetes, hypertension, chronic kidney disease, cardiovascular disease, vitamin B12 deficiency or nutritional anemia, and autoimmune diseases, residual confounding cannot be completely excluded. Detailed medication exposure, including neurotoxic drugs, cumulative dose, duration, and adherence, was not consistently available. Fourth, detection bias should be considered. Participants with thyroid diseases may have more frequent healthcare contact, laboratory testing, or hospital visits, increasing the likelihood of peripheral neuropathy being diagnosed and recorded. We were unable to comprehensively adjust for healthcare utilization in the current analysis. Fifth, the null association observed for thyroid cancer should be interpreted cautiously because the number of participants with thyroid cancer was relatively small, and limited statistical power or type II error cannot be excluded. Finally, the UK Biobank cohort is not fully representative of the general population, as participants are generally healthier, have higher socioeconomic status, and are less ethnically diverse than the broader population. Therefore, generalizability to other populations may be limited.

In conclusion, in this large population-based cohort, both hypothyroidism and hyperthyroidism were associated with higher odds of peripheral neuropathy. These findings provide further evidence of an association between thyroid diseases and peripheral neuropathy. Further studies are needed to better understand the underlying mechanisms and the clinical implications of these associations.

## Data Availability

The data analyzed in this study is subject to the following licenses/restrictions: The UK Biobank data used in this study are available through application to the UK Biobank and can be accessed by qualified researchers following approval of a research proposal. Requests to access these datasets should be directed to https://www.ukbiobank.ac.uk/.
