# Supplementary material for: Thyroid diseases and risk of peripheral neuropathy in a large population-based cohort: evidence from the UK Biobank
Source: Front Endocrinol (Lausanne). 2026 May 19;17:1828737. doi: 10.3389/fendo.2026.1828737 (PMC13226562; doi:10.3389/fendo.2026.1828737)
Supplement: Supplementary file 1 [file Table1.docx]

**Supplementary Table S1. ICD-10 codes and definitions used to identify peripheral neuropathy in the study**

| ICD-10 code | Definition |
| --- | --- |
| G60 | Hereditary and idiopathic neuropathy |
| G61 | Inflammatory polyneuropathy |
| G62 | Other polyneuropathies |
| G63 | Polyneuropathy in diseases classified elsewhere |
| G64 | Other disorders of peripheral nervous system |
